# Long COVID among Brazilian Adults and Elders 12 Months after Hospital Discharge: A Population-Based Cohort Study

**DOI:** 10.3390/healthcare12141443

**Published:** 2024-07-19

**Authors:** Maria Aparecida Salci, Lígia Carreira, Natan Nascimento Oliveira, Natan David Pereira, Eduardo Rocha Covre, Giovanna Brichi Pesce, Rosana Rosseto Oliveira, Carla Franciele Höring, Wanessa Cristina Baccon, Jesús Puente Alcaraz, Giovana Alves Santos, Ludmila Lopes Maciel Bolsoni, Andrés Gutiérrez Carmona, João Ricardo Nickenig Vissoci, Luiz Augusto Facchini, Carlos Laranjeira

**Affiliations:** 1 Departamento de Pós-Graduação em Enfermagem, Universidade Estadual de Maringá, Avenida Colombo, 5790, Campus Universitário, Maringá 87020-900, PR, Brazil; masalci@uem.br (M.A.S.); ligiacarreira.uem@gmail.com (L.C.); pg55215@uem.br (N.N.O.); naatan_daviid@hotmail.com (N.D.P.); eduardocovre@hotmail.com (E.R.C.); gipesce@gmail.com (G.B.P.); rosanarosseto@gmail.com (R.R.O.); wanessa.baccon@gmail.com (W.C.B.); giovanaalves497@gmail.com (G.A.S.); ludmilalopesbolsoni@gmail.com (L.L.M.B.); 2 Departamento de Estatística, Universidade Estadual de Maringá, Avenida Colombo, 5790, Campus Universitário, Maringá 87020-900, PR, Brazil; cfhoring2@uem.br; 3 Department of Health Science, University of Burgos, Paseo de los Comendadores, s/n, 09001 Burgos, Spain; jpalcaraz@ubu.es; 4 Departamento de Enfermería, Universidad de Antofagasta, #02800, Antofagasta 1270300, Chile; andres.gutierrez.carmona@uantof.cl; 5 Emergency Medicine Division, Department of Surgery, Duke University, Durham, NC 27708, USA; jnv4@duke.edu; 6 Division of Global Neurosurgery and Neurology, Department of Neurosurgery, Duke Global Health Institute, Duke University, Durham, NC 27708, USA; 7 Departamento de Medicina Social, Faculdade de Medicina e Programa de Pós-Graduação em Epidemiologia e Saúde da Família e Programa de Pós-Graduação em Enfermagem, Universidade Federal de Pelotas, Pelotas 96010-610, RS, Brazil; luizfacchini@gmail.com; 8 School of Health Sciences, Polytechnic University of Leiria, Campus 2, Morro do Lena, Alto do Vieiro, Apartado 4137, 2411-901 Leiria, Portugal; 9 Centre for Innovative Care and Health Technology (ciTechCare), Polytechnic University of Leiria, Campus 5, Rua das Olhalvas, 2414-016 Leiria, Portugal; 10 Comprehensive Health Research Centre (CHRC), University of Évora, 7000-801 Évora, Portugal

**Keywords:** COVID-19, epidemiology, prevalence studies, Long COVID, signs and symptoms, Brazil

## Abstract

The persistence of symptoms for more than three months following infection with severe acute respiratory syndrome coronavirus 2 is referred to as “Long COVID”. To gain a deeper understanding of the etiology and long-term progression of symptoms, this study aims to analyze the prevalence of Long COVID and its associated factors in a cohort of Brazilian adults and elders, twelve months after hospital discharge. An observational, prospective, and follow-up study was performed with a cohort of adults and older adults diagnosed with COVID-19 in 2020 in the State of Paraná, Brazil. Twelve months after hospital discharge, patients answered a phone questionnaire about the persistence of symptoms after three levels of exposure to COVID-19’s acute phase (ambulatory, medical ward, and intensive care unit). According to the characteristics of participants, the prevalence of Long COVID-19 was calculated, and logistic regression analyses were conducted. We analyzed data from 1822 participants (980 adults [≥18–<60 years] and 842 older people [≥60 years]) across three exposure levels. The overall Long COVID prevalence was 64.2%. Long COVID was observed in 646 adults (55%; of which 326 were women) and 523 older people (45%; of which 284 were women). Females had a higher prevalence of long-term symptoms (52%) compared with men. The most common post-COVID-19 conditions in the 12-month follow-up were neurological (49.8%), followed by musculoskeletal (35.1%) and persistent respiratory symptoms (26.5%). Male individuals were less likely to develop Long COVID (aOR = 0.50). Other determinants were also considered risky, such as the presence of comorbidities (aOR = 1.41). Being an adult and having been hospitalized was associated with the development of Long COVID. The risk of developing Long COVID was twice as high for ward patients (aOR = 2.53) and three times as high for ICU patients (aOR = 3.56) when compared to non-hospitalized patients. Presenting clinical manifestations of digestive (aOR = 1.56), endocrine (aOR = 2.14), cutaneous (aOR = 2.51), musculoskeletal (aOR = 2.76) and psychological systems (aOR = 1.66) made adults more likely to develop Long COVID. Long COVID was present in a large proportion of people affected by the SARS-CoV-2 infection. Presence of Long COVID symptoms displayed a dose–response relationship with the level of disease exposure, with a greater prevalence of symptoms associated with the severe form in the acute period.

## 1. Introduction

The spread of SARS-CoV-2 and its repercussions are public health problems of international concern, since the beginning of the pandemic in December 2019. By 19 May 2024, more than 775 million cases of COVID-19 and more than 7 million deaths were confirmed worldwide [1].

The number of individuals who recovered from the disease is expressive. However, the number of individuals with late and lasting symptoms is increasing, resulting in impaired activities beyond the disease’s acute phase [2]. The condition was named ‘Long COVID’ by the UK National Health Service (NHS), which was then used by the National Institute for Health and Care Excellence (NICE) and the World Health Organization (WHO) [3]. Long COVID can be defined as signs and symptoms reported after infection with SARS-CoV-2 and manifested for more than twelve weeks and unrelated to alternative diagnoses [4]. The condition is associated with all age groups and with different degrees of severity of the disease in its acute phase [5,6,7].

Some systematic reviews have reported patients with various systemic manifestations triggered by the disease and evidence of probable repercussions on vital organs [6], which can be extensive and generate numerous negative consequences for the body [8,9,10]. Long COVID can have more intense effects on several systems: respiratory (dyspnea and decreased diffusion capacity); hematological (thrombolytic events and hyper-inflammatory states); cardiovascular (thoracic pain, cardiometabolic alterations and cardiac fibrosis); neuropsychiatric (cognitive impairment, anxiety, depression) [11,12]; renal (reduced glomerular filtration rate); endocrine (worsening metabolic disorders such as diabetes mellitus and thyroiditis); gastrointestinal (change in intestinal microbiota); and dermatological (capillary loss) [13].

Over 100 million individuals worldwide are afflicted with Long COVID. Current knowledge limits our ability to diagnose this illness, predict outcomes, provide treatment, and offer care services [14]. Furthermore, the estimated economic impact of Long COVID is expected to be immense. In the United States alone, a staggering 4 million individuals have been rendered unable to work. The estimated cost ranges from USD 2.6 to 3.7 trillion, including both diminished quality of life and earning capacity [15]. In addition to the incapacity and suffering caused by the condition, people with Long COVID have also experienced denial, stigmatization, and a lack of effective therapies from healthcare practitioners.

After analyzing 130 papers, Woodrow et al. [16] stated that the prevalence of Long COVID (lasting ≥12 weeks) following SARS-CoV-2 infection varied depending on how symptoms were diagnosed and quantified. The estimates ranged from 0% to 93%, with a pooled estimate of 42.1% and a 95% prediction ranging from 6.8% to 87.9%.

Although Long COVID is gaining more attention, there is still a lack of comprehensive research regarding the variables that contribute to its prevalence and severity. Prior studies on Long COVID have provided useful insights into its prevalence and symptomatology, yet there are still ongoing limitations and gaps in our understanding. Brazil registered a significant number of COVID-19 infections—38.8 million confirmed cases by May 2024 [17]; however, there are a lack of studies examining the variables related to Long COVID in Brazil. Therefore, the present study aimed to analyze the prevalence of Long COVID and associated factors in a cohort of Brazilian adults and older people, 12 months after hospital discharge. Identifying the variables linked to Long COVID is imperative to alleviate the impact of this condition. Moreover, identifying the most vulnerable demographics might inform the development of focused interventions, such as vaccination initiatives, for those who are likely to develop Long COVID.

## 2. Materials and Methods

### 2.1. Study Design

This observational, prospective, and follow-up study analyzed a cohort of adults and older people diagnosed with COVID-19 in the State of Paraná (Brazil)—called “Cohort COVID-19 Paraná/UEM”. Primary and secondary data were extracted from public databases, including the Paraná State COVID-19 Notification System (Notifica COVID) and the Influenza Epidemiological Surveillance System (SIVEP-Gripe), and through telephone interviews. A detailed description of the study procedures was published [18].

The study followed the recommendations in Strengthening the Reporting of Observational Studies in Epidemiology (STROBE) [19].

### 2.2. Setting, Sample, and Recruitment

The study involved people aged ≥18 who had COVID-19 in the State of Paraná, Brazil, in 2020. According to the World Health Organization [9], patients with a positive COVID-19 diagnosis (nasopharyngeal swab RT-qPCR) were classified according to the severity of their symptoms: (1) Mild Cases: asymptomatic people or with symptoms not suggestive of viral pneumonia; (2) Moderate Cases: mild signs suggestive of viral pneumonia, including SpO2 ≥ 90% on room air; (3) Severe Cases: the presence of signs suggestive of severe viral pneumonia plus respiratory rate ≥ 30 breaths per minute, dyspnea or SpO2 ≤ 90% on room air; and (4) Critical Cases: persistence of severe pneumonia symptoms for more than a week, development of Severe Acute Respiratory Syndrome, sepsis, septic shock, acute thrombosis or Multisystem Inflammatory Syndrome [9].

Due to the availability of clinical information in databases, participants in this study were classified according to their place of treatment: (1) Mild cases: those who underwent outpatient care, with no need for hospitalization; (2) Moderate cases: patients who required hospitalization in a ward to treat COVID-19 symptoms; and (3) Severe/critical cases: patients who required hospitalization in Intensive Care Units (ICU) or those who underwent invasive mechanical ventilation, regardless of the place of hospitalization, due to the overload of the healthcare system.

The study’s sample size was determined independently for mild–moderate cases and severe cases. Previous estimates assumed a 20% prevalence rate for mild–moderate cases and determined that a sample size of 400 was necessary [20]. To account for severe cases, we assumed Long COVID had a prevalence rate of 50% and predicted that a sample size of 100 would be necessary. Both computations were performed with a relative precision of 20%. Hence, at least 500 participants were needed for the study.

### 2.3. Outcomes

For this study, the presence of persistent symptoms was evaluated 12 weeks after discharge from hospitalization due to COVID-19, a condition known as Long COVID [18,21]. Patients were stratified according to where treatment for COVID-19 occurred (outpatient, medical ward, and ICU) and age group (adults and older adults). Persistent self-reported symptoms were analyzed in two ways, namely: (1) symptoms were grouped by organic system affected: neurological (headache, eye pain, change in vision, change in smell, change in taste, change in speech, change in hearing, ringing in the ears, tingling or numbness throughout the body, dizziness, loss of movement coordination, and memory loss), respiratory (runny nose, sore throat, hoarse voice, cough, phlegm production, chest pain, and shortness of breath), digestive (change in stool, change in appetite, nausea, cramps, and abdominal pain), endocrine (hair loss and perspiration at rest), dermatological (spots on the body and itching), musculoskeletal (problems in muscles and joints and tiredness/fatigue), cardiovascular (edema) and psychological (depression and anxiety); and, (2) presence of each symptom analyzed independently.

### 2.4. Independent Variables

The independent variables related to the outcome included: exposure factor (Outpatient; Medical Ward; ICU), age group (Adults [≥18–<60 years old]; Older Adults [≥60 years old]), sex (Female; Male); race (White; Non-White; Preferred not to answer); and previous diagnosis of chronic non-communicable diseases (Yes; No). The data were obtained from records in the databases and complemented by telephone interviews.

### 2.5. Follow-Up Interview after 12 Months

Research participants were evaluated 12 months after the date of hospital discharge, based on their type of treatment for COVID-19 and age group. These exposures observe the dose–response of the disease and its effect after discharge [18]. Telephone contacts were made after selecting potential participants from public databases, using available telephone contacts. Participants were contacted by a data collection team previously trained by experts to carry out the interviews, guided by a form created for this purpose.

### 2.6. Data Analysis

The data were tabulated in Excel sheets and anonymized. Subsequently, these data were imported into the R software, version 4.3.0, for descriptive analysis, estimation of Relative Risk (RR), and logistic regression to identify predictor variables. Before the analyses, the data were thoroughly evaluated to resolve any missing data and outliers, and to test the assumption of multivariate normality (using the Kolmogorov–Smirnov test). To evaluate the influence of missing data, we conducted our analysis by excluding those cases with any “unknown” variables.

The descriptive analysis was carried out by calculating the absolute and relative frequencies of the variables. Furthermore, the global prevalence of Long COVID was calculated and stratified by age group and exposure factor. Likewise, the Relative Risks (RR) and the Confidence Interval (CI) between Outpatient (control exposure factor, assuming value 0) and Ward and ICU settings (dose–response exposure factors, assuming values 1 and 2, respectively) were estimated for the symptom clusters and individual symptoms.

The RR is the ratio between the population with the exposure factor and presence of Long COVID symptoms (a) divided by the sum of people in the exposure factor (a + c) and the population with the control exposure factor and presence of symptoms (b) divided by the sum of the people in the control exposure factor (b + d) [22], as shown in Table 1.

$$Relative\;Risk\;(RR)=\;\frac{\frac{a}{a+c}}{\frac{b}{b+d}}$$

where:

a is the number of individuals in the Ward/ICU group with persistent symptoms;
b is the number of individuals in the Outpatient group with persistent symptoms;
c is the number of individuals in the Ward/ICU group without persistent symptoms;
d is the number of individuals in the Outpatient group without persistent symptoms.

We estimated the CI for the RR using the Wald method. Symptoms with a CI greater or less than 1.00 were considered significant. The epiR package (Version 2.0.75) was used to perform these analyses [23]. Subsequently, a logistic regression was performed to identify the factors associated with Long COVID assessed 12 months after hospital discharge (outcome variable). Univariate associations were modeled and variables whose *p* < 0.20 were considered for the multivariate analysis. Based on previous research, we computed various models that were adjusted for covariables (age, sex, comorbidities, and race) [24,25]. Multivariate models were adjusted using both-direction stepwise regression and the best-fitting model was identified using the Akaike information criterion (AIC). The interpretations of the univariate and multivariate models were based on the estimation of Odds Ratio (OR) and 95% CI. The presence of multicollinearity was evaluated by calculating the variance inflation factor, whereas the linearity of the logit was examined for continuous variables. Nagelkerke’s R^2^ was utilized to assess the adequacy of model fit for the final multivariable models. For all tests, a *p* < 0.05 was considered significant.

### 2.7. Ethics

The study followed the recommendations proposed by the Declaration of Helsinki and obtained a favorable opinion from the State University of Maringá with nº 4.165.272 and CAAE: 34787020.0.0000.0104. It also received approval from the Hospital do Trabalhador, an agency representing the Health Department of the State of Paraná, through opinion No. 4.214.589 and CAAE: 34787020.0.3001.5225. All participants were informed about the objectives of the study and were asked about their interest in participating in the research. The terms of consent were sent to those who agreed to participate, via email or mail, according to the participant’s request.

## 3. Results

**Figure 1:**
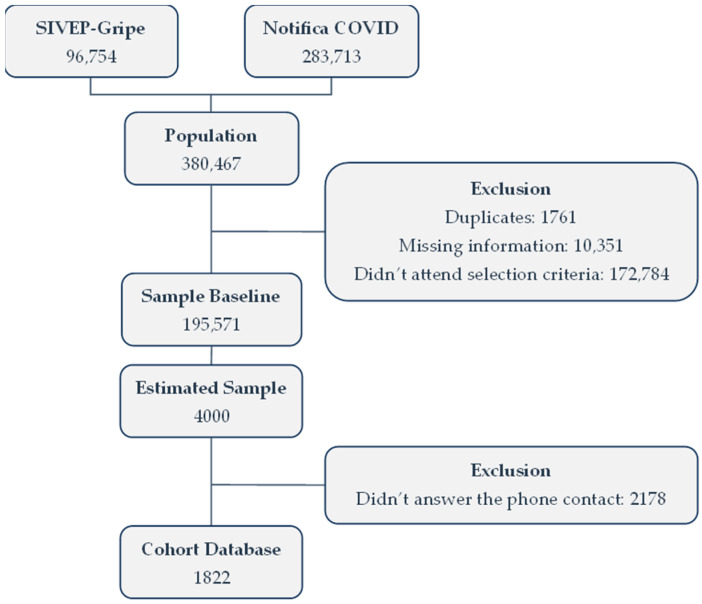
Flow chart of selection of the subjects.

Between January and December 2020, 380,467 individuals with COVID-19 were registered on the platforms. Subsequently, duplicated records, records with missing information, or that did not meet the selection criteria were excluded. Given the resulting baseline for sample calculation, telephone contacts were made. Unsuccessful contacts were excluded, leading to a final study sample consisting of 1822 participants, as shown in Figure 1.

### 3.1. Prevalence of Long COVID among Study Subjects

Of the 1822 participants, 1169 presented self-reported symptoms 12 months after discharge (prevalence = 64.2%). The most common post-COVID-19 conditions in the 12-month follow-up were neurological (49.8%), followed by musculoskeletal (35.1%) and respiratory persistent symptoms (26.5%). The highest prevalence of Long COVID was observed in patients who had developed the most severe cases. Patients who had been hospitalized in the ICU (38%) were the most affected, followed by those hospitalized in wards (31%) or treated in outpatient settings (31%). Data from 980 adults and 842 older people were analyzed. Long COVID was observed in 646 adults (55%) and 523 older people (45%). While most participants were male, females had a higher prevalence of long-term symptoms (52%). Furthermore, the highest prevalence of Long COVID was observed in white participants (49%) and those diagnosed with chronic non-communicable diseases (63%) (Table 2).

Table 3 describes the sociodemographic characteristics of the studied population, according to age group and type of treatment for COVID-19. Male participants had a higher prevalence of ICU hospitalization in both adult (59%) and elderly (50%) groups. Women were mostly treated as outpatients, both in the adult group (67%) and among older people (59%). Regarding race, in adults, hospitalization in the ward was more prevalent among white individuals (59%). Among white older people, ICU admissions were more prevalent (57%). The diagnosis of chronic non-communicable diseases also influenced the type of treatment, depending on the age group. Among adults diagnosed with chronic non-communicable diseases, hospitalizations were more prevalent in the ICU (62%), a fact that also affects the older group (76%).

Table 4 presents the symptomatic manifestations of Long COVID, in adults and older people according to the place of treatment. The symptoms were divided by bodily systems. In adults hospitalized in a ward and who developed Long COVID, the most prevalent neurological changes included vision changes (RR = 2.43), memory loss (RR = 1.69), and tingling or numbness in some parts of the body (RR = 2.15); respiratory changes included chest pain (RR = 1.99) and shortness of breath (RR = 1.56); and, digestive changes included changes in appetite (RR = 2.19) and nausea (RR = 5.58). There were also endocrine changes, such as sweating at rest (RR = 2.13); skin changes, such as spots on the body (RR = 9.81); musculoskeletal changes, evidenced by tiredness/fatigue (RR = 1.93) and problems in muscles and joints (RR = 1.83); and psychological changes, with the occurrence of depression (RR = 1.98) and anxiety (RR = 1.66).

In adult patients with Long COVID who required hospitalization in the ICU, prevalent neurological changes included vision changes (RR = 3.52), changes in speech (RR = 2.55), dizziness (RR = 2.94), loss of coordination of movements (RR = 6.33), memory loss (RR = 1.86) and tingling or numbness in some part of the body (RR = 4.81); respiratory changes were evidenced by runny nose (RR = 4.23), hoarse voice (RR = 3.33), chest pain (RR = 2.15) and shortness of breath (RR = 2.36); digestive changes included changes in appetite (RR = 2.55); skin changes were evidenced by spots on the body (RR = 10.17) and body itching (RR = 15.26); musculoskeletal manifestations included tiredness/fatigue (RR = 2.18) and problems in muscles and joints (RR = 2.43); cardiovascular changes included edema (RR = 4.57); and psychological manifestations included depression (RR = 2.34) and anxiety (RR = 1.64).

In the case of older people with Long COVID who were hospitalized in a ward, prevalent neurological changes were loss of movement coordination (RR = 2.34) and memory loss (RR = 1.14); endocrine changes included hair loss (RR = 1.61); musculoskeletal changes were evidenced by tiredness/fatigue (RR = 1.66); and psychological changes were manifested by reports of anxiety (RR = 1.77).

As for older patients hospitalized in the ICU and who developed Long COVID, there was significant prevalence of neurological changes, such as headache (RR = 2.38), vision changes (RR = 1.79), changes in smell (RR = 2.10), change in speech (RR = 3.33), dizziness (RR = 1.75), loss of coordination of movements (RR = 3.99), memory loss (RR = 1.90) and tingling or numbness in some part of the body (RR = 2.61). Among the respiratory changes, hoarse voice (RR = 5.08), chest pain (RR = 2.72), and shortness of breath (RR = 2.83) were significant. Digestive changes were also evident, such as changes in appetite (RR = 2.33) and changes in feces (RR = 2.85). Furthermore, there were endocrine changes, with hair loss (RR = 2.33), and skin changes, such as spots on the body (RR = 4.76) and body itching (RR = 2.51). Musculoskeletal changes were evidenced by the presence of tiredness/fatigue (RR = 2.19) and problems with muscles and joints (RR = 1.93). Cardiovascular changes, such as edema (RR = 2.53), and psychological changes, such as depression (RR = 2.41) and anxiety (RR = 2.50), were also reported (Table 4).

### 3.2. Factors Associated with Long COVID

Adults presented several predictive factors for the occurrence of Long COVID (Table 5). Male individuals were less likely to develop the condition (aOR = 0.50), while other determinants were considered risky, such as the presence of comorbidities (aOR = 1.41). Regarding the type of treatment, hospitalized adults were associated with the development of Long COVID. Ward patients had twice the risk (aOR = 2.53) and ICU patients three times the risk (aOR = 3.56) of developing Long COVID, when compared to outpatients. Regarding acute symptoms of COVID-19, adults with clinical manifestations in the digestive (aOR = 1.56), endocrine (aOR = 2.14), cutaneous (aOR = 2.51), muscular (aOR = 2.76) and psychological systems (aOR = 1.66) were more likely to develop Long COVID. The model was able to explain 36.4% of the total variance in long COVID-19 (Nagelkerke’s R^2^ = 0.364; *p* < 0.001).

In the older group, the predictors of Long COVID included having been diagnosed with comorbidities (aOR = 1.83), having been hospitalized in the ICU (aOR = 2.46), and with acute manifestations of COVID-19, particularly digestive (aOR = 1.81), endocrine (aOR = 1.74), skin (aOR = 2.46), and muscular (aOR = 2.45) changes. Unlike adults, older people had an increased risk of Long COVID if they had presented cardiovascular manifestations during the acute phase (aOR = 3.59). The model predicted 29.7% of the total variance in Long COVID (Nagelkerke’s R^2^ = 0.297; *p* < 0.001).

## 4. Discussion

This study aimed to analyze the prevalence of Long COVID and associated factors in a cohort of adults and older people in Southern Brazil. Like previous studies investigating post-COVID-19, we used a telephone survey [26,27]. The results indicate that more severe cases of COVID-19 are more likely to lead to persistent symptoms after the infection’s acute phase. This study unveiled a substantial prevalence of Long COVID: 64.2% of those recuperating from COVID-19. Other studies in Brazil revealed higher prevalences (67.5% [28]; 77.4% [29]).

In the study sample, men tended to have more severe COVID-19 in the acute phase, justifying admission to the ICU. Recent studies demonstrated that men have an increased risk of developing a more severe condition from COVID-19, suggesting the influence of biological, sociocultural, and behavioral factors intrinsic to sex [30,31]. Other population studies carried out in China [32] and Europe [33] showed similar data, with men having more severe cases.

In the analysis adjusted for the development of Long COVID, men had a 50% lower chance of developing this condition compared to women, suggesting that women may have a greater susceptibility to this condition. Women have faster and more robust adaptive immune responses, which can protect them from the initial infection and its severity [34]. However, this same difference can make women more vulnerable to long-term illnesses, such as autoimmune-related diseases [35]. This was confirmed in this study, where female sex and other comorbidities were strongly related to Long COVID [36,37,38]. In parallel, research carried out in Brazil shows that women were less likely to seek medical care for COVID-19-related symptoms than men [39]. Due to pandemic constraints, women assumed the primary responsibility for childcare “hindering their ability to seek health care for themselves” [39] (p. 2208).

In our cohort, white individuals predominated. Evidence shows that black patients have a worse evolution of COVID-19 and greater mortality risk, compared to non-black individuals [40]. Racial and ethnic minorities have experienced increased morbidity and mortality from COVID-19 and tend to possess socioeconomic factors and comorbidities associated with worse COVID-19 outcomes, such as obesity and hypertension [41,42,43].

The results of this study indicate that the severity of the disease, as expressed by the place of treatment (ICU or medical ward for adults and ICU for older people), is related to a significant increase in the risk of developing Long COVID. This finding is consistent with prior data [44,45,46]. Our results suggest that comorbidities may be associated with a greater risk of developing Long COVID in adults and older people. Furthermore, most individuals hospitalized in the ICU had previous chronic conditions, which may indicate they were more likely to develop serious forms of the disease [47,48]. Evidence indicates that a wide range of comorbidities (such as fibromyalgia, depression, anxiety and celiac disease) are associated with an increased risk of COVID-19 symptoms for up to twelve weeks after infection [49]. The relationship between the use of ventilatory support and the development of Long COVID was also considered. The results indicate that most patients hospitalized in the ICU showed residual lung changes over up to 6 months, regardless of whether ventilatory support was used or not, but total lung capacity was lower in those treated with invasive ventilation [50].

Furthermore, and corroborating previous evidence [2,51,52,53,54,55], our results showed symptomatologic manifestations of Long COVID in adults and older people, depending on the location of COVID-19 treatment and the division of signs and symptoms by body systems. The results also indicate that COVID-19 organ-specific symptoms during the acute phase (i.e., digestive, endocrine, cutaneous, musculoskeletal and cardiovascular problems in adults and older people) are associated with a greater risk of developing Long COVID. In addition to these symptoms, older adults were also more likely to develop cardiovascular symptoms and the adults were more at risk of developing psychological problems associated with Long COVID.

The literature shows that patients who received outpatient treatment, who had mild symptoms of COVID-19, may also present post-acute and prolonged symptoms of the disease. A systematic review suggests that the frequency of persistent symptoms in patients with mild infection varies between 10% and 35%, and they can be distinguished into physical, mental, and social symptoms. Fatigue is the most frequently described persistent neurological symptom, ranging from 30% to 87%, with a prevalence of 0.23 [2]. Furthermore, neurological problems such as memory loss, cognitive disorders, paresthesia, sleep disorders, musculoskeletal pain, and dizziness are widely evidenced with high prevalence, ranging from 25% to 52% [2,54,55,56,57].

Other frequently occurring long-term symptoms are dyspnea, cough, chest pain, headache, decreased mental and cognitive status, and olfactory dysfunction. Furthermore, persistent symptoms after a mild COVID-19 infection can have important consequences for work and daily functioning [2].

In this study, memory loss after COVID-19 was a significant symptom among adults and older people, occurring more frequently with more severe cases of the disease. A cohort study in South America identified that the memory and attention of people who had COVID-19 significantly decreased when compared to people without a history of infection [58]. Of the 36 symptoms evaluated in this research, the least prevalent was vomiting, both for adults and older people, a result similar to other studies [59]. These findings emphasize the significance of considering the medical history of COVID-19 patients when assessing the likelihood of Long COVID. Based on current results, the experience of Long COVID disrupts the lives of people suffering from this condition. Given the impact of Long COVID, affected people need to reorganize their routines, develop adaptive strategies, and access specialized health services whenever necessary [60,61].

### 4.1. Study Limitations

There were several limitations in our study. Firstly, the use of epidemiological surveillance systems to track possible contacts can influence the selection of study participants, compromising the representativeness of the sample and outcomes. We also collected acute data from the electronic medical records without accessing additional information that could potentially be obtained from the medical notes. To address any potential inaccuracies in the demographic information reported by the patients during hospital admission, we verified this information during follow-up telephone interviews. This is a recognized and prevalent constraint with cohorts formed using this methodology. Secondly, as calls were made by telephone, numerous contacts may have been misinterpreted as telemarketing, causing a negative reception by respondents, and negatively influencing our response rates. Furthermore, considering the difficulty of conducting remote interviews, the questions were asked succinctly and objectively to improve understanding by the interviewees, but thereby limiting the depth of the interviews and fostering response homogeneity. Despite the many variables under study, other variables such as COVID-19 vaccination coverage should be analyzed in further research. We acknowledge that face-to-face interviews and/or objective measurement of symptoms would deliver more robust results. Moreover, the lack of a control group and an intermediate follow-up time point complicates the ability to identify any discernible patterns of improvement or deterioration. Furthermore, our study did not include an evaluation of specialist medical services such as psychological or psychiatric care, nor did it consider diagnostic services. Therefore, the actual magnitude of healthcare service consumption may be underestimated.

In Brazil, the rate of underreporting of COVID-19 was alarming, especially considering that healthy individuals with symptoms consistent with COVID-19 were instructed to stay at home without seeking medical attention [62]. Furthermore, throughout the progression of the COVID-19 pandemic, there was a dearth of screening tests offered by the Brazilian Unified Health System, as well as a limited supply of intensive care unit (ICU) beds for individuals with severe infections [62]. The current study did not estimate the frequency of underreporting or the number of undetected cases of COVID-19. This could potentially affect prevalence estimates [63] and compromise the generalizability of our results to broader populations affected by COVID-19. Further research should consider individuals with milder or asymptomatic infections as a key aspect in the fight against infection by SARS-CoV-2.

Despite the limitations associated with telephone surveys, these were necessary due to financial and pandemic restrictions. We reinforce the importance of future research expanding the number of participants and sampling other regions of the country and the world, seeking to understand the impact of Long COVID. Furthermore, the need to include validated instruments and the importance of conducting interviews in both modalities (remote and in-person) are confirmed.

Despite these limitations, our study presents relevant data on the health implications of COVID-19 survivors and reinforces the need for other studies that longitudinally monitor these individuals.

### 4.2. Implications for Practice

The results of this study have relevant implications for healthcare practice in the face of the long-term effects of COVID-19. The correlation identified between the initial severity of the disease and the incidence of Long COVID highlights the need for preventive measures, appropriate treatments, and careful monitoring of patients facing more serious cases. The relevance of this study transcends the clinical scope and is essential to guide public policies aimed at the management and prevention of prolonged cases of the disease.

This study also highlights the need for an integrated and collaborative approach to healthcare practice, recognizing the complexity and variety of manifestations of Long COVID [64]. Understanding the factors that contribute to this prolonged condition can inform more effective treatment and support strategies. The most recent evidence suggests gains associated with cardiopulmonary rehabilitation in patients with Long COVID, indicating this strategy should be generalized [65]. As we move forward, future research can delve deeper into the chronic nature of COVID-19, offering important insights for public health and directing broader social policies. Addressing the challenges associated with Long COVID requires a holistic, collaborative and future-oriented approach that integrates continuous learning and responds to community and global health needs.

## 5. Conclusions

To our knowledge, this is one of the largest cross-sectional prevalence studies of Long COVID-19 among Brazilian patients hospitalized with COVID-19 that includes data from a 12-month follow-up. The overall prevalence of Long COVID was 64.2%. This suggests that infected people have a significant need for specialist services that cater to the needs of people suffering from post-COVID-19 syndrome.

In this study, the results suggest that Long COVID can be characterized, in adults, by a higher prevalence of memory loss, shortness of breath, change in appetite, hair loss, spots on the body and itching, tiredness/fatigue, edema, depression, and anxiety. Furthermore, in older people, the most prevalent symptoms were memory loss, lack of appetite change, hair loss, body itching, tiredness/fatigue, edema, depression, and anxiety. The long-term effects of COVID-19, across the population, should be a priority for further research to define Long COVID, its risk factors and underlying causes, and to formulate strategies for prevention, rehabilitation, and public health actions to improve recovery and long-term outcomes. Several crucial aspects of Long COVID research are emphasized, such as the significance of drawing insights from previous studies on post-viral and post-infection diseases, the necessity for adequate funding for Long COVID research, and the importance of collaboration and communication among stakeholders (i.e., researchers, healthcare providers, and patients). It is also imperative to prioritize low-income countries and more vulnerable populations, who lack sufficient information regarding Long COVID. Lastly, utilizing multidisciplinary and patient-centered care in long-term follow-ups can help design customized interventions, as standard diagnostic procedures do not capture the complexity of Long COVID.

## Figures and Tables

**Table 1:** Exposure factors for calculating the Prevalence Ratio.

	Exposure Factor 1/2	Exposure Factor 0	Total
Persistent symptom +	a	b	a + b
Persistent symptom −	c	d	c + d
Total	a + c	b + d	a + b + c + d

**Table 2:** Sociodemographic and in-hospital characteristics of the sample stratified by the presence of Long COVID (N = 1822).

Variables	Long COVID		*p* †
	No, n = 653	Yes, n = 1169	
Age group			0.091
Adult	334 (51%)	646 (55%)	
Older Adult	319 (49%)	523 (45%)	
Sex			**<0.001**
Female	270 (41%)	610 (52%)	
Male	383 (59%)	559 (48%)	
Race			0.200
White	228 (35%)	573 (49%)	
Non-White	139 (21%)	296 (25%)	
Preferred not to answer	286 (44%)	300 (26%)	
Previous Diagnosis of Non-communicable Disease			**<0.001**
No	374 (57%)	435 (37%)	
Yes	279 (43%)	734 (63%)	
Type of treatment for COVID-19			**<0.001**
Outpatient	379 (58%)	364 (31%)	
Medical Ward	164 (25%)	365 (31%)	
ICU	110 (17%)	440 (38%)	

† *p*-value of the Chi-square test for the categorical variables. Bold means *p* < 0.05.

**Table 3:** Sociodemographic characteristics of the sample stratified by age and type of treatment for COVID-19 (N = 1822).

Variables	Adults				Older Adults			
	Outpatient (n = 187)	Medical Ward (n = 201)	ICU (n = 249)	*p*	Outpatient (n = 177)	Medical Ward (n = 155)	ICU (n = 191)	*p*
Sex				**<0.001**				0.213
Female	125 (67%)	98 (47%)	103 (41%)		105 (59%)	83 (54%)	96 (50%)	
Male	62 (33%)	112 (53%)	146 (59%)		72 (41%)	72 (46%)	95 (50%)	
Race				0.053				0.170
White	80 (43%)	119 (59%)	109 (44%)		82 (46%)	74 (48%)	109 (57%)	
Non-white	59 (32%)	50 (25%)	68 (27%)		44 (25%)	38 (24%)	37 (19%)	
Preferred not to answer	48 (25%)	41 (20%)	72 (29%)		51 (29%)	43 (28%)	45 (24%)	
Non-communicable Chronic Disease				**<0.001**				0.566
No	114 (61%)	90 (43%)	94 (38%)		50 (28%)	42 (27%)	45 (24%)	
Yes	73 (39%)	120 (57%)	155 (62%)		127 (72%)	113 (73%)	146 (76%)	

ICU: Intensive Care Unit. Bold means *p* < 0.05.

**Table 4:** Persistent symptoms of the sample stratified by age and type of treatment for COVID-19 (N = 1822).

Persistent Symptoms	Adults (n = 980)					Older Adults (n = 842)				
	Outpatient (n = 392)	Medical Ward (n = 279)		ICU (n = 309)		Outpatient (n = 351)	Medical Ward (n = 250)		ICU (n = 241)	
	Prev.	Prev.	RR (CI 95%)	Prev.	RR (CI 95%)	Prev.	Prev.	RR (CI 95%)	Prev.	RR (CI 95%)
Neurological persistent symptoms	36.6	54.6	1.49 (1.26–1.76)	66.1	1.81 (1.55–2.10)	36.0	42.3	1.18 (0.96–1.44)	63.4	1.76 (1.49–2.09)
Headache	7.37	11.00	1.49 (0.93–2.40)	10.03	1.36 (0.85–2.19)	3.27	5.17	1.58 (0.74–3.36)	7.78	2.38 (1.18–4.78)
Eye pain	2.19	3.77	1.72 (0.72–4.10)	4.02	1.84 (0.80–4.25)	3.27	3.27	1.00 (0.43–2.34)	4.71	1.44 (0.66–3.15)
Change in vision	5.13	1250	2.43 (1.45–4.08)	18.07	3.52 (2.18–5.67)	7.97	9.26	1.16 (0.70–1.94)	14.29	1.79 (1.13–2.85)
Change in smell	10.32	8.62	0.84 (0.52–1.34)	9.4	0.91 (0.58–1.42)	5.99	7.69	1.28 (0.72–2.29)	12.60	2.10 (1.25–3.53)
Change in taste	7.65	7.22	0.94 (0.55–1.61)	9.38	1.22 (0.76–1.98)	6.85	8.76	1.28 (0.75–2.19)	8.95	1.31 (0.76–2.25)
Change in speech	1.69	2.39	1.41 (0.50–3.98)	4.32	2.55 (1.04–6.24)	1.63	2.90	1.77 (0.62–5.05)	5.45	3.33 (1.30–8.56)
Change in hearing	2.67	3.40	1.27 (0.55–2.96)	4.01	1.50 (0.68–3.31)	3.27	5.47	1.67 (0.80–3.52)	10.16	3.11 (1.60–6.04)
Ringing in the ears	2.92	4.42	1.51 (0.70–3.27)	4.00	1.37 (0.63–2.96)	3.27	7.69	2.35 (1.18–4.70)	7.81	2.39 (1.19–4.80)
Dizziness	3.17	6.16	1.94 (0.97–3.90)	9.32	2.94 (1.56–5.54)	6.03	5.47	0.91 (0.48–1.72)	10.55	1.75 (1.02–3.00)
Loss of coordination of movements	2.18	3.75	1.72 (0.72–4.10)	13.79	6.33 (3.14–12.77)	3.27	7.64	2.34 (1.17–4.66)	13.04	3.99 (2.10–7.57)
Memory loss	24.40	41.20	1.69 (1.36–2.11)	45.30	1.86 (1.50–2.29)	22.60	25.80	1.14 (0.86–1.51)	42.90	1.90 (1.50–2.41)
Tingling or numbness throughout the body	3.65	7.85	2.15 (1.14–4.05)	17.55	4.81 (2.77–8.34)	6.61	5.47	0.83 (0.44–1.55)	17.25	2.61 (1.63–4.18)
Respiratory persistent symptoms	16.0	27.0	1.69 (1.26–2.26)	37.2	2.32 (1.78–3.03)	16.4	24.3	1.48 (1.08–2.03)	38.3	2.33 (1.76–3.09)
Runny nose	0.73	1.71	2.34 (0.56–9.71)	3.09	4.23 (1.17–15.24)	2.19	3.65	1.67 (0.67–4.16)	4.67	2.13 (0.88–5.14)
Sore throat	1.94	2.72	1.40 (0.53–3.69)	1.85	0.95 (0.33–2.71)	1.64	2.54	1.55 (0.53–4.55)	2.71	1.66 (0.56–4.87)
Hoarse voice	1.95	2.38	1.22 (0.45–3.34)	6.48	3.33 (1.49–7.42)	1.91	3.64	1.91 (0.74–4.95)	9.69	5.08 (2.23-11.57)
Cough	3.92	6.90	1.76 (0.93–3.33)	7.21	1.84 (0.99–3.42)	4.68	4.78	1.02 (0.50–2.07)	12.11	2.59 (1.46–4.57)
Phlegm production	1.46	3.08	2.11 (0.76–5.87)	3.38	2.32 (0.87–6.20)	3.01	2.55	0.84 (0.33–2.15)	8.53	2.83 (1.40–5.73)
Chest pain	3.43	6.83	1.99 (1.02–3.87)	7.38	2.15 (1.13–4.09)	2.73	2.92	1.07 (0.43–2.67)	7.42	2.72 (1.28–5.74)
Shortness of breath	11.2	17.5	1.56 (1.08–2.25)	26.6	2.36 (1.70–3.28)	8.22	13.5	1.64 (1.04–2.59)	23.23	2.83 (1.88–4.26)
Digestive persistent symptoms	4.96	10.65	2.15 (1.25–3.69)	12.26	2.47 (1.47–4.15)	8.56	10.19	1.19 (0.73–1.94)	15.75	1.84 (1.18–2.86)
Change in appetite	3.45	7.56	2.19 (1.14–4.21)	8.81	2.55 (1.37–4.77)	5.19	7.72	1.49 (0.82–2.71)	12.11	2.33 (1.35–4.04)
Nausea	0.49	2.72	5.58 (1.19–26.08)	1.54	3.15 (0.62–16.15)	0.27	1.09	4.02 (0.42-38.42)	1.95	7.14 (0.84-60.75)
Vomiting	0.00	0.00	-	0.00	-	0.00	0.37	-	0.00	-
Cramps or abdominal pain	1.21	1.36	1.12 (0.30–4.14)	2.14	1.76 (0.57–5.51)	2.19	0.73	0.33 (0.07–1.56)	3.89	1.78 (0.71–4.44)
Change in stool consistency	1.46	2.38	1.63 (0.56–4.81)	2.77	1.90 (0.68–5.29)	1.91	2.56	1.34 (0.48–3.78)	5.45	2.85 (1.17–6.96)
Endocrine persistent symptoms	20.0	25.7	1.28 (0.97–1.69)	25.7	1.29 (0.98–1.69)	11.8	16.2	1.37 (0.92–2.03)	23.9	2.02 (1.41–2.90)
Hair loss	17.9	20.9	1.17 (0.86–1.59)	20.8	1.16 (0.86–1.58)	9.55	15.38	1.61 (1.05–2.47)	22.22	2.33 (1.57–3.45)
Perspiration at rest	2.38	7.82	2.13 (1.13–4.01)	6.13	1.67 (0.87–3.21)	3.01	1.81	0.60 (0.21–1.71)	4.96	1.56 (0.70–3.48)
Cutaneous persistent symptoms	0.24	3.07	12.62 (1.61–99.11)	4.98	20.49 (2.73-53.66)	3.83	2.94	0.77 (0.33–1.81)	10.51	2.75 (1.47–5.13)
Spots on the body	0.24	2.38	9.81 (1.21–79.30)	2.47	10.17 (1.28-80.92)	0.82	0.36	0.45 (0.05–4.27)	3.89	4.76 (1.32-17.13)
Body itching	0.24	1.03	4.23 (0.44–40.49)	3.7	15.26 (1.99-16.74)	3.56	2.55	0.72 (0.29–1.77)	8.95	2.51 (1.30–4.87)
Musculoskeletal persistent symptoms	21.6	40.7	1.88 (1.49–2.38)	46.2	2.14 (1.71–2.67)	22.5	33.6	1.49 (1.15–1.93)	46.2	2.05 (1.62–2.59)
Tiredness/Fatigue	19.1	36.9	1.93 (1.50–2.49)	41.5	2.18 (1.71–2.77)	18.2	30.2	1.66 (1.25–2.21)	39.9	2.19 (1.68–2.87)
Muscle and joint problems	6.59	12.03	1.83 (1.13–2.95)	16.03	2.43 (1.56–3.79)	9.97	11.9	1.19 (0.76–1.87)	19.29	1.93 (1.30–2.88)
Cardiovascular persistent symptoms	-	-	-	-	-	-	-	-	-	-
Edema	1.70	4.10	2.41 (0.96–6.05)	7.76	4.57 (2.00–10.43)	3.27	3.27	1.00 (0.43–2.34)	8.27	2.53 (1.27–5.05)
Psychological persistent symptoms	12.6	21.9	1.74 (1.24–2.44)	25.3	2.02 (1.46–2.78)	9.44	14.93	1.58 (1.03–2.43)	25.20	2.67 (1.81–3.92)
Depression	7.65	15.12	1.98 (1.28–3.05)	17.92	2.34 (1.55–3.54)	7.16	10.74	1.50 (0.90–2.49)	17.27	2.41 (1.52–3.82)
Anxiety	10.90	18.20	1.66 (1.15–2.41)	17.90	1.64 (1.14–2.36)	6.06	10.7	1.77 (1.04–3.00)	15.14	2.50 (1.52–4.12)

Prev.: Prevalence; RR: Relative Risk; CI: Confidence Interval; ICU: Intensive Care Unit.

**Table 5:** Unadjusted and adjusted regression models of factors associated with Long COVID (N = 1822).

Variables	Adults				Older Adults			
	Unadjusted Model		Adjusted Model		Unadjusted Model		Adjusted Model	
	OR (95% CI)	*p*	aOR (95% CI)	*p*	OR (95% CI)	*p*	aOR (95% CI)	*p*
Sex								
Female	Reference	-	Reference	-	Reference		Reference	-
Male	0.60 (0.47–0.78)	<0.001	0.50 (0.36–0.67)	**<0.001**	0.74 (0.57–0.97)	0.027	0.80 (0.59–1.08)	0.200
Comorbidities								
No	Reference		Reference	–	Reference		Reference	–
Yes	2.28 (1.76–2.96)	<0.001	1.41 (1.04–1.92)	**0.029**	2.53 (1.91–3.34)	<0.001	1.83 (1.34–2.51)	**<0.001**
Type of treatment								
Outpatient	Reference		Reference		Reference		Reference	
Medical Ward	3.02 (2.20–4.17)	<0.001	2.53 (1.74, 3.69)	**<0.001**	1.38 (1.01–1.88)	<0.001	1.15 (0.81–1.63)	0.400
ICU	3.86 (2.81–5.33)	<0.001	3.56 (2.46–5.19)	**<0.001**	3.06 (2.17–4.34)	<0.001	2.46 (1.67–3.63)	**<0.001**
Symptoms of Acute COVID-19								
Neurological	3.17 (2.16–4.69)	<0.001	–	–	2.86 (2.05–4.00)	<0.001	–	–
Respiratory	3.71 (2.68–5.18)	<0.001	–	–	3.47 (2.54–4.77)	<0.001	–	–
Digestive	3.82 (2.94–4.99)	<0.001	1.56 (1.12–2.18)	**0.008**	3.76 (2.85–4.97)	<0.001	1.81 (1.30–2.51)	**<0.001**
Endocrine	4.62 (3.40–6.37)	<0.001	2.14 (1.47–3.13)	**<0.001**	3.70 (2.64–5.25)	<0.001	1.74 (1.17–2.59)	**0.006**
Cutaneous	5.68 (2.97–12.3)	<0.001	2.51 (1.23–5.68)	**0.017**	5.93 (3.07–12.9)	<0.001	2.46 (1.17–5.86)	**0.027**
Musculoskeletal	5.98 (4.53–7.94)	<0.001	2.76 (1.98–3.85)	**<0.001**	4.19 (3.16–5.57)	<0.001	2.45 (1.77–3.39)	**<0.001**
Cardiovascular	6.38 (2.95–16.7)	<0.001	–	–	7.59 (3.51–19.9)	<0.001	3.59 (1.46–10.9)	**0.011**
Psychological	3.57 (2.71–4.72)	<0.001	1.66 (1.19–2.32)	**0.003**	3.15 (2.36–4.22)	<0.001	–	–

OR: Odds Ratio; aOR: Adjusted Odds Ratio; CI: Confidence Interval. Bold means *p* < 0.05.

## Data Availability

The data are available upon reasonable request.
